# The Impact of DRG-Based Payment Reform on Inpatient Healthcare Utilization: Evidence from a Natural Experiment in China

**DOI:** 10.3390/healthcare13192424

**Published:** 2025-09-24

**Authors:** Hua Zhang, Xin Fu, Yuhan Wu, Yao Tang, Hui Jin, Bo Xie

**Affiliations:** 1 Department of Health Insurance, School of Public Health, Southeast University, Nanjing 210009, China; wuyuhan0120@163.com; 2 Department of Epidemiology and Health Statistics, School of Public Health, Southeast University, Nanjing 210009, China; xwz_9421@163.com; 3 Department of Psychology, Dornsife College of Letters, Arts and Science, University of Southern California, Los Angeles, CA 90089, USA; tytang@usc.edu; 4 Department of General Medicine, School of Medicine, Southeast University, Nanjing 210009, China; boxxie80@163.com

**Keywords:** diagnosis-related groups, healthcare quality and efficiency, health policy reform

## Abstract

**Objectives**: This study aims to examine the impact of Diagnosis-Related Group (DRG) payment on medical costs, efficiency, and quality of healthcare services in public hospitals, providing policy recommendations for further health insurance payment reforms in China. **Methods**: Utilizing inpatient medical insurance settlement data from 2020 to 2023 in the selected city, we constructed a regression discontinuity design (RDD) and an interrupted time series (ITS) model to evaluate the causal effects of the DRG reform. The analysis includes 66,533 inpatient settlement records. **Results**: Following the reform, the average length of stay (LOS) decreased by 2 days (95% CI: −3.43 to −0.70, *p* < 0.01), total hospitalization expenditures dropped by 13% (95% CI: −0.26 to −0.00, *p* < 0.05), and expenditures from the medical insurance fund declined by 25% (95% CI: −0.39 to −0.12, *p* < 0.01). Additionally, examination and consultation fees were reduced by 23% (95% CI: −0.41 to −0.05, *p* < 0.05), although patients’ out-of-pocket burden increased by 8% (95% CI: 0.05 to 0.10, *p* < 0.01). In terms of healthcare quality, the 30-day readmission rate decreased by 1% (95% CI: −0.01 to −0.00, *p* < 0.01), and the mortality rate among low-risk patients declined by 4% (95% CI: −0.04 to −0.03, *p* < 0.01). We found no evidence of patient selection or denial of admission. Heterogeneity analysis revealed that the reduction in hospital stay was concentrated among enrollees under the Urban and Rural Resident Basic Medical Insurance and those treated in secondary hospitals. The policy’s effects peaked shortly after implementation but gradually attenuated over time. **Conclusions**: Our study offers hospital-level evidence indicating that the initial stage of DRG implementation achieved its preliminary goals of optimizing medical resource allocation and improving the efficiency of medical insurance fund utilization. However, the reform still faces several challenges. These findings may offer valuable references for developing countries pursuing reforms in primary healthcare and health insurance payment systems.

## 1. Introduction

Before the 1990s, China’s hospital payment system primarily relied on fee-for-service (FFS) retrospective payments. This approach created perverse incentives, such as over-treatment, excessive medical expenditure, and irrational pricing behaviors, which contributed to the disorderly expansion of the healthcare market. Both public and private healthcare expenditures soared, at times exceeding GDP growth rates [1]. In response, the Chinese government launched a new round of healthcare reforms in 2009, aiming to provide safe, efficient, and affordable basic healthcare services to the population. Since then, basic health insurance coverage has steadily exceeded 95% [2,3]. However, amid slowing economic growth, an aging population, and rising healthcare demands, the healthcare system faces mounting challenges, including inefficiencies, unsustainable expenditures, and increasing public dissatisfaction [4]. Thus, it is imperative to explore more effective models of governance, organizational, and management and to improve the use and allocation of medical insurance funds to deliver higher quality and more efficient hospital services.

The concept of Diagnosis-Related Groups (DRG) was first introduced in the late 1960s in the United States as a mechanism to control healthcare costs more effectively. DRGs classify patients into clinically and economically comparable groups based on diagnosis, severity, and complications. Under this system, payments are based on the group assigned after diagnosis rather than the actual costs incurred during treatment. While DRG systems have been widely studied and applied across a variety of healthcare systems worldwide over the past few decades, robust empirical evidence on their effectiveness in developing countries remains limited, and the impacts across different medical institutions and insured populations are still debated [5,6,7,8].

Z City, located in eastern China, had a per capita GDP of CNY 183,000 and a population of 9.547 million in 2023. On 1 January 2022, Z City officially launched DRG payment reform for all secondary and tertiary healthcare institutions (excluding psychiatric hospitals). The reform applied to patients enrolled in both the Urban Employee Basic Medical Insurance (UEBMI) and the Urban-Rural Resident Basic Medical Insurance (URRBMI), creating a natural exogenous policy shock.

This study utilized inpatient data for colorectal cancer patients in Z City from 2020 to 2023 to quantitatively evaluate the impact of DRG payment reform on medical outcomes. It also examined heterogeneity in effects across insurance types and hospital levels, aiming to inform evidence-based policymaking to optimize health insurance payment mechanisms and promote more equitable and efficient healthcare resource allocation.

This study is organized as follows: Section 2 presents the contributions relative to previous studies and the research questions. In Section 3, we introduce the data collection and methodology used. Section 4 provides the results. In Section 5, we provide an interpretation and discussion of the results. And then discuss research implications and limitations. The conclusions are presented in Section 6.

## 2. Background

### 2.1. The Chinese DRG System

As the world’s largest developing country, China faces rising medical expenditures and inefficiencies in healthcare delivery. In response, a comprehensive reform of the medical insurance payment system is underway. In 2009, the State Council issued the “Opinions on Deepening the Reform of the Medical and Health System,” which set the overarching goal of establishing a basic medical and healthcare system that covers both urban and rural residents, ensuring access to safe, effective, convenient, and affordable care. Before this reform, most public hospitals in China operated under FFS payment models, which fueled uncontrolled growth in healthcare costs and incentivized hospitals to overprovide services for additional revenue, leading to widespread over-treatment and inefficiency [9]. With the onset of population aging, these structural problems increasingly challenges the short- and long-term financial sustainability of China’s healthcare insurance system [4,10].

China began experimenting with DRGs in the 1980s. In 2008, the first domestic DRG classification system—BJ-DRG was developed mainly based on the U.S. and Australian models [11]. Since then, regional governments have piloted and refined DRG systems. In 2017, the State Council issued further guidelines to promote medical insurance payment reform, mandating DRG-based payment pilots and encouraging alternatives such as capitation to replace traditional FFS approaches [12]. After more than two decades of experimentation and development, four main DRG variants emerged: BJ-DRG (focused on payment, used in 12 provinces), CN-DRG (focused on performance and quality, used in 29 provinces), CR-DRG (targeting the New Rural Cooperative Medical Scheme and urban-rural resident programs), and C-DRG (piloted by the National Health Commission in cities like Sanming and Karamay) [13]. In October 2019, China introduced its first unified DRG system, CHS-DRG, which integrates the BJ, CN, and CR versions [14]. Implementation followed a three-stage process—top-level design, simulated operation, and full implementation in 30 cities’ pilot programs. By leveraging DRG, China aims to shift from retrospective to prospective payment models, realign the interests of hospitals, insurers, and patients, and ultimately improve the efficiency of insurance fund utilization. The DRG framework also facilitates payment negotiations between healthcare institutions and insurers, supports financial balance, incentivizes clinical staff, standardizes medical practices, improves service efficiency, and promotes the sustainable development of the healthcare system [15].

As an early adopter of China’s Diagnosis-Related Groups (DRG) payment reform, Z City has extended DRG-based payments to all 98 of its secondary-level and above medical institutions since 2022. This city-wide reform encompasses all inpatient care categories, characterized by extensive coverage, substantial scale of implementation, and a high degree of data transparency. Key strengths of Z City’s DRG model include standardized policy enforcement and exceptional data transparency. The healthcare insurance bureau releases operational indicators—including weight adjustments, Case Mix Index (CMI), and cost structures—on a quarterly basis, providing researchers with high-quality longitudinal datasets. Moreover, Z City implemented the reform simultaneously across all institutions from the outset, avoiding potential selection bias associated with staggered rollout. This natural experiment setting offers a robust opportunity to evaluate the causal effects of DRG policies on treatment practices for specific diseases.

### 2.2. Existing Research and Evidence

As the continuous rise in medical costs poses severe challenges to the healthcare security system, the Diagnosis-Related Group (DRG) payment reform has become a crucial direction for China’s healthcare policy reform. Existing research primarily unfolds from two dimensions: On one hand, a large number of studies based on macro data have confirmed that DRG plays a positive role in controlling the growth of medical expenses, shortening average hospitalization days, and optimizing medical resource allocation [16,17]; On the other hand, some scholars have begun to focus on the impact of DRG on clinical diagnosis and treatment behaviors, finding that it may induce medical institutions to adjust patient admission structures and lead to phenomena such as diagnostic code upgrades [18]. In recent years, the academic community has gradually extended its research focus to specific disease areas, analyzing the impact of DRG on medical quality [19]. These studies provide important references for understanding the policy effects of DRG.

However, current research still faces significant limitations. First, for colorectal cancer—a highly heterogeneous malignancy with complex treatment protocols—systematic evaluations of DRG payment reform’s impact on clinical practices, cost structures, and healthcare quality remain lacking. Second, existing studies predominantly employ policy before-after comparisons or cross-sectional analyses, which fail to adequately control for temporal trends and other confounding factors, potentially introducing bias in policy effect estimation. More importantly, while City Z has achieved full regional coverage, synchronized implementation, and high data transparency in its DRG pilot program, creating an ideal natural experiment setting for rigorous policy evaluation, no study has fully utilized these conditions to apply causal inference methods like Regrettable Difference (RDD) for identifying DRG’s net effects on colorectal cancer outcomes. Therefore, leveraging City Z’s reform practices and RDD methodology to scientifically assess DRG’s influence on colorectal cancer treatment not only helps fill existing research gaps but also provides empirical evidence and policy references for expanding DRG adoption in complex disease management.

## 3. Methodology

### 3.1. Dataset

This study investigates the impact of DRG payment reform on public hospitals from the perspectives of medical expenditure, efficiency, and quality. Colorectal cancer, characterized by high global incidence and mortality rates, entails complex treatment pathways and substantial healthcare costs. Accordingly, we utilize inpatient data of colorectal cancer patients from City Z, China, spanning the years 2020 to 2023. Based on the coverage scope of relevant policies, records from primary healthcare institutions, psychiatric hospitals, and cross-regional hospitalizations were excluded. The final sample comprises 66,533 inpatients covered by basic medical insurance.

The selection of outcome variables is informed by the Performance Evaluation Manual for Tertiary Public Hospitals (2023 Edition) published by the National Health Commission of China, along with statistical indicators from the World Health Organization and the World Bank. The length of stay (LOS) is used as the primary indicator of healthcare efficiency. Medical expenditure variables include total expenditure, medical costs paid by medical insurance, out-of-pocket, and expenditures on medications, diagnostics, and consumables. Healthcare quality was measured using the 30-day readmission rate and the mortality rate among low-risk patients. All cost variables are reported in nominal CNY according to the year of hospitalization. We did not adjust expenditures to constant prices because the study period (2020–2023) was relatively short and inflation in medical services was modest.

Control variables include demographic characteristics (e.g., age, gender), institutional attributes (e.g., hospital type, hospital grade, tertiary-A classification), type of medical insurance, and the age-adjusted Charlson Comorbidity Index (CCI). To address heteroscedasticity in regression analysis, all expenditure-related variables are log-transformed using the natural logarithm. To reflect the severity of illness, we adopt the age-adjusted CCI, where higher index scores indicate greater disease severity. Given that disease severity also varies with age, we further adjust the CCI according to age groups using the method proposed by Quan [20] (see Appendix A). Table 1 summarizes and illustrates the variables used in this study.

In defining the low-risk mortality indicator, patients with pathological stage I or II and a CCI score ≤ 1 are classified as low-risk. Considering that severely ill patients with extended hospital stays and high medical costs are not suitable for DRG-based payments, we excluded extreme outliers in both hospitalization days and costs. Additionally, 1% winsorization was applied to the sample for regression analysis. All statistical analyses were performed using Stata/MP version 17.0 (StataCorp LLC, College Station, TX, USA).

Table 2 presents the summary statistics of the main variables in our study. A total of 66,533 colorectal cancer inpatients from City Z between 2020 and 2023 were included as the study sample. The average age of the patients was 65 years, with a predominance of male patients, accounting for 62.69%, which aligns with the known epidemiological characteristics of colorectal cancer. Regarding insurance coverage, 51,285 patients (77.08%) were enrolled in the Urban Employee Basic Medical Insurance (UEBMI) scheme, while 15,248 patients (22.92%) were covered under the Urban and Rural Resident Basic Medical Insurance (URRBMI) scheme. Most patients received treatment in general hospitals, with a high concentration in tertiary care institutions, which constituted 87.44% of the sample. Among these, tertiary-A hospitals accounted for 80.39%, indicating that the majority of patients were treated in high-level healthcare facilities.

### 3.2. Analysis Strategy

Since the 1990s, regression discontinuity design (RDD) has gradually become one of the key methods for policy evaluation. Given that the probability of adopting the new payment reform in Z City changed completely from 0 to 1, it meets the criteria for a sharp regression discontinuity design. Therefore, this study uses the policy implementation date as the cutoff point and constructs the following regression discontinuity model for empirical analysis:

Y_i_ = α + β_1_D_i_ + f(d_i_,D_i_) + β_2_X_i_ + ε_i_ (−h ≤ d_i_ ≤ h) (1)

where Y_i_ is the dependent variable such as LOS, expenditures, readmission, mortality and so on. And D_i_ is the treatment group indicator. The running variable d represents the difference between the settlement date and the policy implementation date. The model includes a polynomial function of both the running variable and the treatment indicator. Specifically, D = 1 when d ≥ 0, and D = 0 when d < 0. The coefficient β captures the local average treatment effect at the cutoff, which is the primary estimate of the policy impact. X_i_ denotes a vector of control variables, and standard errors are clustered at the hospital level. H is bandwidth window around the cutoff, selected by mean squared error minimization. ε_i_ is error term, clustered at the hospital level. This study primarily adopts a non-parametric estimation approach, applying a triangular kernel weighting function. The optimal bandwidth is selected by minimizing the mean squared error (MSE). For robustness checks, the results of linear parametric regressions are also reported.

To examine the Changes in trends associated with the policy reform, this study employs an Interrupted Time Series (ITS) analysis to estimate both the immediate and sustained effects of the reform. Using 1 January 2022 as the intervention point, an ITS regression model is constructed to analyze the trend in the length of hospital stay before and after the implementation of the DRG-based payment reform. Given that the policy took effect immediately, the model does not account for any time lag in the response. A single-group ITS analysis is conducted using monthly aggregated data, with the regression model specified as follows:

Y_i_ = β_0_ + β_1_ × time_i_ + β_2_ × intervention_i_ + β_3_ × timeafterintervention_i_ + ε_i_ (2)

Y_i_ represents the length of hospital stay. β_0_ denotes the estimated initial level of the outcome variable during the observation period. β_1_ captures the estimated pre-reform trend in the outcome variable, serving as the baseline slope. β_2_ reflects the estimated immediate change in the outcome variable at the point of policy intervention, while β_3_ represents the difference in the slope of the outcome trend before and after the reform. εi denotes the random error term. The Newey-West method is applied to address potential autocorrelation in the error terms.

RDD focuses on local causal effects around the cutoff, while ITS captures broader temporal dynamics. By combining these methods, we provide a more robust assessment of policy impact.

## 4. Results

### 4.1. Description of Variables

Table 3 summarizes the changes in key study variables before and after the implementation of the DRG-based payment system. Following the reform, medical costs paid by medical insurance funds decreased significantly by 610.54 CNY (*p* < 0.01), while out-of-pocket expenditures by patients increased by 758.44 CNY (*p* < 0.01). Medicine costs declined, whereas costs on diagnostics and consumables exhibited an upward trend. These findings suggest that the reform contributed positively to cost containment, reflecting an enhanced awareness of cost-saving practices among healthcare providers. In addition, the reduction in LOS indicates improved healthcare efficiency. Regarding healthcare quality, both the 30-day readmission rate and the low-risk mortality rate decreased after the reform, by 3% and 9% respectively, suggesting potential improvements in clinical outcomes associated with DRG-based payment.

### 4.2. Regression Discontinuity Results

**Figure 1:**
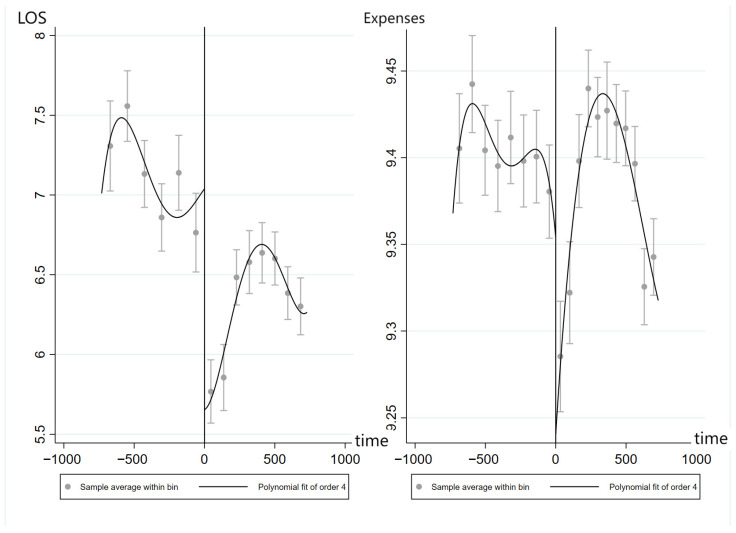
Discontinuity plots of LOS (**Left**) and expenses (**Right**) pre- and post-DRG reform.

Before conducting regression analysis, it is necessary to test whether there is a significant discontinuity in the outcome variables around the cutoff point of the DRG payment reform. As shown in Figure 1, there is a clear jump in the length of hospital stay at the cutoff point of the reform, indicating that the reform has a reducing effect on hospital stay duration. Similarly, the right panel of the figure displays a discontinuous change in healthcare expenditures, suggesting a significant shift.

It is evident that before and after the reform, average cost per admission, total hospitalization costs, reimbursement from the medical insurance fund, and patients’ out-of-pocket expenses all exhibit significant discontinuities.

#### 4.2.1. Impacts of DRG on Medical Costs and LOS

Table 4 presents the estimation results of the Regression Discontinuity (RD) model assessing the impact of the payment reform on medical expenditures and healthcare efficiency. The findings indicate that following the introduction of the DRG-based payment system, both total hospitalization costs and medical insurance fund expenditures exhibited a downward trend. Specifically, total expenditure decreased by 13% (95% CI: −0.26 to −0.00, *p* < 0.05), while insurance fund payments declined by 25% (95% CI: −0.39 to −0.12, *p* < 0.01). Conversely, the patient out-of-pocket payment rate increased by 8% (95% CI: 0.05 to 0.10, *p* < 0.01). Among expenditure components, diagnostic examination costs within hospitalization expenses decreased by 23% (95% CI: −0.41 to −0.05, *p* < 0.05). Although medicine and consumable costs also declined, these reductions were not statistically significant. In addition to cost-related outcomes, the DRG reform led to a reduction in average LOS by approximately 2 days (95% CI: −3.43 to −0.70, *p* < 0.01), suggesting improvements in healthcare efficiency.

#### 4.2.2. Impacts of DRG on Medical Service Quality

This study employed the 30-day readmission rate and low-risk mortality rate as indicators of healthcare quality. Table 5 presents the 30-day readmission rate decreased by 1% (95% CI: −0.01 to −0.00, *p* < 0.01), and the low-risk mortality rate declined by 4% (95% CI: −0.04 to −0.03, *p* < 0.01), indicating that the DRG-based payment reform had a positive effect on quality-related outcomes.

In addition, we examined the impact of the reform on CCI, which accounts for the presence of 19 predefined comorbid conditions. We hypothesized that if patient selection bias were present, the implementation of DRG payment might lead to a reduction in CCI scores. However, the results showed no statistically significant causal relationship between the DRG reform and changes in CCI (95% CI: −0.03 to 0.04, *p* = 0.86). This suggests that patient selection bias was not a concern in this study.

### 4.3. Interrupted Time Series (ITS) Analysis

The analysis revealed that the immediate effect of the reform on LOS was statistically significant, with an estimated coefficient (β_2_) of −0.8 days (95% CI: −1.31 to −0.32, *p* < 0.01), indicating a notable reduction immediately following the implementation of the DRG-based payment system. However, the post-reform trend coefficient (β_3_) was 0.05 (95% CI: 0.02 to 0.09, *p* < 0.01), suggesting a gradual increase in LOS over time. These results imply that while the reform initially contributed to a reduction in hospitalization duration, the long-term effect appears to be attenuated, with LOS gradually returning to an upward trajectory.

### 4.4. Heterogeneity Analysis

#### 4.4.1. Different Insured Populations

In addition, subgroup analyses were conducted for individuals enrolled in UEBMI and URRBMI. As shown in Table 6, a total of 50,366 inpatient settlement records were obtained for UEBMI participants and 14,818 for URRBMI participants. The results indicate that the DRG-based payment reform had a more pronounced impact on LOS for URRBMI beneficiaries. Specifically, the LOS decreased by 2.2 days (95% CI: −3.95 to −0.47, *p* < 0.05) for URRBMI patients, compared to a 1.87-day reduction (95% CI: −3.22 to −0.53, *p* < 0.01) among UEBMI patients.

Table 6 shows the insurance fund was more substantial among URRBMI patients, with a 37% reduction (95% CI: −0.58 to −0.16, *p* < 0.01). Correspondingly, the out-of-pocket for this group increased by 15% (95% CI: 0.11 to 0.18, *p* < 0.01), Nevertheless, the overall financial burden remained relatively comparable. In terms of healthcare quality, no adverse effects were observed in either insurance group following the implementation of the reform.

#### 4.4.2. Different Classification of Hospital (COH)

Subsequently, the analysis was stratified by hospital level, distinguishing between secondary and tertiary hospitals, with additional focus on Grade III-A hospitals, as presented in Table 7. The results show that the majority of patients were treated in tertiary hospitals, while only 8190 cases were recorded in secondary hospitals. Among tertiary hospitals, Grade III-A institutions accounted for a substantial proportion.

The reform’s impact on controlling total hospitalization expenditures and insurance fund spending was primarily observed in tertiary hospitals. Specifically, after the implementation of the DRG-based payment reform, total hospitalization expenditures in tertiary hospitals decreased by 14% (95% CI: −0.28 to −0.00, *p* < 0.01), whereas no statistically significant effects were detected in secondary hospitals.

Across all hospital types, the proportion of out-of-pocket increased to varying degrees. The average LOS declined by 1 to 3 days post-reform, with a more pronounced reduction observed in secondary hospitals. Importantly, no adverse effects were found on the quality-of-care indicators in any hospital category.

### 4.5. Robustness Test

#### 4.5.1. Premise Assumption

**Figure 2:**
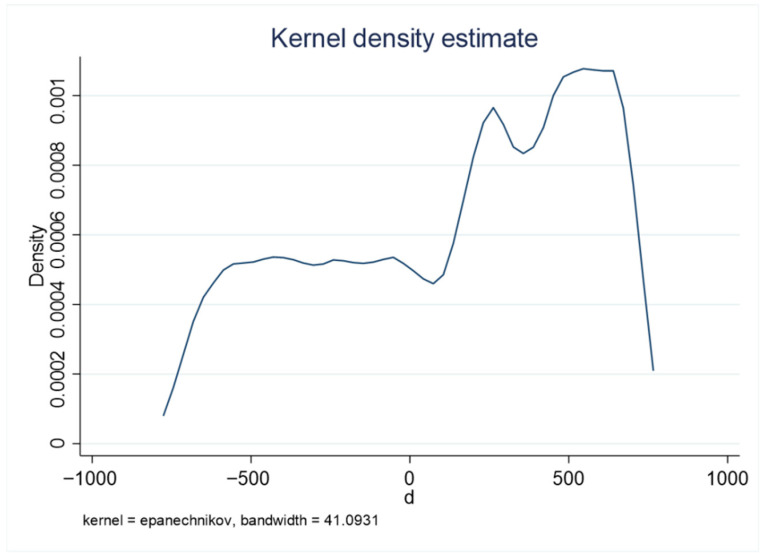
Density histogram of settlement time.

The validity of the regression discontinuity design (RDD) relies on two key assumptions. First, the distribution of the sample around the cutoff point should be random, meaning there is no self-selection bias; individuals should not be able to subjectively manipulate their assignment to different groups. In the context of this study, the driving variable is the patient’s settlement time, and the policy reform is an exogenous natural shock with mandatory implementation. Whether a patient is affected by the DRG payment system reform depends solely on whether their settlement time falls before or after the reform and cannot be influenced by the patient’s or healthcare provider’s subjective choices. Therefore, self-selection bias is not a concern in this study. Figure 2 presents the density histogram of settlement times, which does not show any significant discontinuous changes.

The second assumption is the continuity test. Near the cutoff point, the distribution of control variables, which do not include treatment effects, should be continuous, without any discontinuous jumps. Table 8 presents the conditional densities of various control variables at the cutoff point, and the results show that most are not statistically significant, indicating the absence of discontinuities.

#### 4.5.2. Sensitivity Analysis of Bandwidth

The choice of bandwidth is typically a critical setting in regression discontinuity design (RDD). If the bandwidth is too large, the similarity of samples on either side of the cutoff point decreases, leading to an increased bias in the estimate of the average treatment effect. However, this allows for more observations to be included, which can improve the accuracy of the estimates. Conversely, a smaller bandwidth reduces the bias but may lower the accuracy of the estimates. To test the sensitivity of the results to the choice of bandwidth, this study conducts regressions with bandwidths set at 0.8 and 1.2 times the optimal bandwidth, as presented in Table 9. The results show that the conclusions remain largely consistent, indicating that the findings are robust.

#### 4.5.3. Parameter Estimation

To validate the robustness of the nonparametric test results, this study also employs parametric estimation. Specifically, a second-order linear parametric regression using a matrix kernel function is implemented. As shown in Table 10, the results are broadly consistent with those obtained from the nonparametric analysis, further supporting the reliability of the findings.

Additionally, the above regressions were conducted after removing all control variables, and the results remained largely unchanged (see Table 11). A placebo test was also performed by randomly selecting pseudo-cutoffs to simulate alternative breakpoints. The majority of the results were not statistically significant (see Table 12).

#### 4.5.4. Removing COVID-19 Effects

To account for the potential confounding influence of the COVID-19 pandemic on hospital utilization and expenditures, we re-estimated the models after excluding all observations from the year 2020. As shown in Table 13, the results remained consistent with the main analysis in both magnitude and direction, confirming that the observed effects of DRG reform were not driven by pandemic-related shocks.

## 5. Discussion

### 5.1. The Impact of DRG Reform on the Structure of Medical Expenditure

Data and textual analyses indicate a significant reconfiguration in the distribution of healthcare resource utilization following the implementation of the DRG-based payment system. In particular, the relative proportion of pharmaceutical spending has exhibited a consistent downward trend, whereas the expenditure shares allocated to diagnostic services and medical consumables have shown a gradual increase. To align total healthcare outlays with the expenditure ceilings embedded within the DRG reimbursement framework, clinical practitioners have adopted more stringent prescribing behaviors, actively reducing the use of non-essential medications. This shift is plausibly associated with concurrent systemic policy measures—such as centralized drug procurement programs—that have significantly compressed the profit margins historically linked to pharmaceutical sales. As a result, hospitals have shifted away from drug-centered revenue models, leading to a more structurally balanced allocation of medical expenditure components [21].

Nonetheless, the downward adjustment in pharmaceutical disbursements has been accompanied by a corresponding rise in the financial burden associated with diagnostic interventions and consumable materials, suggesting a potential reorientation in institutional charging practices. This observed redistribution underscores the imperative for healthcare financing authorities to recalibrate regulatory mechanisms and extend supervision to these increasingly salient expenditure domains.

### 5.2. The Impact of DRG Reform on Medical Expenditures

The introduction of DRG-based payment mechanisms has played a significant role in incentivizing hospitals to control healthcare costs. Under the constraint of fixed payment, hospitals are required to optimize resource allocation, avoid unnecessary interventions, and eliminate inefficient or redundant services.

Empirical analysis demonstrates that DRG implementation significantly influences various categories of medical expenditures. Notably, it has led to a measurable decrease in total hospitalization costs and reduced the financial burden on the pooled social insurance fund. These outcomes underscore the reform’s effectiveness in curbing excessive medical spending, avoiding overtreatment, and safeguarding the sustainability of the insurance pool.

However, the observed increase in patients’ out-of-pocket spending raises concerns. This trend may be attributable to the relatively severe disease profiles treated post-reform and to provider behaviors such as recommending non-reimbursable drugs or services to compensate for lost revenues. Such dynamics underscore the importance of strengthening oversight of provider practices and aligning hospital incentives with the broader goals of payment reform [22]. To this end, policymakers should consider expanding the reimbursement scope of the pooled fund and adjusting copayment ratios to prevent undue financial burdens on patients, thereby enhancing the mutual aid function of the insurance system [23]. In parallel, reforms should strengthen catastrophic health insurance mechanisms by offering additional subsidies for high-cost conditions, thus preventing cost-shifting to vulnerable patients.

Heterogeneity analyses indicate that cost reductions are concentrated in tertiary hospitals and among urban employee insurance beneficiaries. Therefore, differentiated incentive mechanisms should be designed to tailor reform strategies to institutional and population-specific contexts [24].

Moreover, the current study is limited to a single disease category within one city, which may not reflect the reform’s broader impact. The effectiveness of DRG payment reforms in controlling costs can vary across healthcare systems. In more developed regions, hospitals benefit from stronger managerial capabilities and higher levels of digital infrastructure, allowing them to leverage DRG mechanisms to improve efficiency and reduce overall expenditures. Conversely, in under-resourced areas, the implementation of DRG reforms may face significant barriers, including poor data quality and limited information systems, making it difficult for payment standards to reflect real-world treatment costs accurately. In such contexts, reforms may inadvertently compromise care quality. Hence, a comprehensive and context-sensitive evaluation of cost-containment outcomes is essential [25].

In conclusion, while DRG-based payment systems hold promise for healthcare cost containment, their ultimate effectiveness hinges on the nuanced details of implementation, such as the appropriateness of payment benchmarks, the administrative capacity of healthcare providers, and the robustness of information systems. Ultimately, cost containment should not be viewed as the end goal of reform. Instead, DRG-based payment systems should serve as managerial instruments and policy levers to guide rational resource allocation and facilitate value-based, precision-oriented healthcare management.

### 5.3. The Impact of DRG Reform on Healthcare Efficiency

The implementation of DRG payment reform has demonstrated considerable potential in enhancing healthcare delivery efficiency by restructuring provider payment mechanisms. Central to the DRG approach is the use of prospective, case-based payments that incentivize healthcare institutions to optimize service provision and reduce unnecessary utilization of medical resources. In contrast to traditional FFS models—where provider revenue is positively correlated with the volume of procedures and services, often resulting in overutilization and inefficiency—the DRG model establishes predetermined payment rates, thereby encouraging providers to deliver care within budgetary constraints. This mechanism promotes the adoption of streamlined clinical pathways, minimizes redundant diagnostics and treatments, and contributes to more efficient inpatient care management [26].

Moreover, DRG reform has catalyzed innovation in hospital administration and altered the prevailing incentive structures within healthcare delivery. Faced with tighter financial margins, hospitals are prompted to improve internal management, refine clinical workflows, and strengthen oversight of both service quality and expenditure. These reforms also necessitate enhanced data governance and greater investment in health information systems, fostering greater transparency and accountability in care delivery. Under such a payment regime, healthcare institutions must not only ensure clinical effectiveness but also design cost-effective therapeutic regimens and optimize bed utilization—ultimately reducing average length of stay (LOS) and enhancing overall system efficiency. Notably, the adoption of DRG systems has contributed to the institutionalization of more scientific and standardized performance assessment mechanisms, with average LOS increasingly regarded as a critical indicator of resource consumption and service efficiency.

Nonetheless, realizing the full efficiency potential of DRG-based payment requires overcoming several structural and operational challenges. Chief among them is the need to establish equitable and clinically appropriate reimbursement rates that reflect the true cost of care delivery, particularly for complex or high-risk patient populations. Failure to do so may inadvertently compromise care quality or discourage providers from admitting patients with more severe conditions. Additionally, designing nuanced payment models that balance cost containment with care adequacy remains essential to avoid under-provision and prevent unintended consequences, such as care avoidance or premature discharge. Therefore, the success of DRG reform is contingent not only upon sound policy design but also upon coordinated efforts from government regulators, healthcare institutions, and other stakeholders to support system-wide transformation.

Finally, the ITS analysis reveals an immediate reduction in average LOS following DRG implementation, suggesting short-term efficiency gains. However, this trend gradually reverses over time, highlighting the need for sustained regulatory oversight to mitigate policy fatigue and preserve the long-term efficacy of reform. Continuous monitoring, periodic adjustment of regulatory instruments, and the introduction of supplementary policy measures will be essential to ensure that DRG reforms yield durable improvements in healthcare efficiency and cost control.

### 5.4. The Impact of DRG Reform on Healthcare Quality

Empirical evidence suggests that the implementation of DRG payment reform has not compromised the quality of care, despite its emphasis on cost containment and efficiency enhancement. Notably, observed reductions in 30-day readmission rates and mortality rates among low-risk patients indicate that the reform has achieved initial success in promoting value-based care without sacrificing clinical outcomes [22]. Furthermore, the absence of patient selection behavior among hospitals—such as rejecting or avoiding less profitable cases—demonstrates that providers have not pursued short-term financial gains at the expense of equitable patient access [27].

This outcome may reflect the effectiveness of regulatory oversight by health insurance authorities in China, who have implemented mechanisms to influence provider behavior within a reasonable range while maintaining healthcare quality. Quality indicators have been incorporated into routine performance evaluations and integrated into the assessment frameworks of public hospitals, forming part of a broader accountability system. These efforts serve to continuously monitor clinical practice, reinforce professional standards among healthcare workers, and deter unethical or non-compliant behaviors.

However, the long-term impact of DRG reform on healthcare quality warrants further investigation. As the reform matures, it is essential to ensure that payment mechanisms continue to incentive quality improvement alongside efficiency. Future iterations of the reform must prioritize the design of clinically appropriate reimbursement standards and strengthen quality assurance systems to guard against unintended adverse effects. Sustained monitoring and adaptive regulation will be critical to ensure that hospitals remain focused on patient-centered outcomes, even as they strive for operational efficiency and cost control.

Ultimately, the success of DRG-based payment reform depends not only on its ability to curb excessive spending but also on its capacity to drive improvements in care delivery. When effectively designed and regulated, DRG reform can serve as a catalyst for hospitals to optimize clinical pathways, enhance management practices, and deliver higher-quality care at a sustainable cost.

### 5.5. Limitations of the Study

This study has several limitations that should be acknowledged. First, The study focus on colorectal cancer patients in a single city (Z City) limits generalizability. Although the sample size is relatively adequate, it lacks horizontal comparisons across different pilot cities and disease types. As a result, the conclusions drawn are context-specific and may not be fully applicable to other diseases or regions. Future research should include multi-site and multi-disease analyses to enhance the external validity and compare with more findings from other diseases or regions, thereby providing more comprehensive evidence for policy evaluation. Second, methodological scope. This study relied on quasi-experimental econometric approaches, namely regression discontinuity design (RDD) and interrupted time series (ITS). While these methods provide credible causal inference in the absence of randomized experiments, they are limited in handling complex nonlinear relationships and high-dimensional interactions. Recent advances in machine learning (e.g., Random Forest, Gradient Boosting) and interpretability tools (e.g., SHAP, LIME) offer promising avenues for capturing heterogeneity in treatment effects and improving predictive performance. Future studies could consider employing more rigorous research designs, such as quasi-experimental approaches or natural experiments, and incorporate data from control cities to generate stronger empirical evidence or integrate econometric and machine learning approaches to strengthen both inference and prediction. Third, choice of outcome measures. We used length of stay (LOS) as the primary indicator of efficiency and defined quality of care based on 30-day readmission and low-risk mortality. These measures are widely applied in health services research and supported by policy practice in China. However, they inevitably capture only certain aspects of efficiency and quality. Other important dimensions, such as patient-reported outcomes, complication rates, and longer-term survival, were not available in our dataset. Future research could incorporate richer quality indicators to provide a more comprehensive evaluation.

### 5.6. Implications for Payment Reform and Future Research

This study provides empirical support for the effectiveness of DRG-based payment reforms in improving hospital efficiency and controlling medical costs without compromising care quality. The observed reductions in length of stay, medical expenditures, and readmission and mortality rates suggest that DRG implementation can enhance resource utilization and promote value-based care. However, the increase in patients’ out-of-pocket burden and the attenuation of policy effects over time highlight the need for continuous policy refinement and monitoring.

For future payment reform, policymakers should consider strategies to mitigate the financial burden on patients while sustaining cost-control incentives for providers. Differentiated policy designs may be necessary to account for variations across insurance types and hospital tiers, as evidenced by the heterogeneity in outcomes. Moreover, the gradual weakening of reform effects underscores the importance of institutionalizing performance evaluation mechanisms and introducing adaptive policy tools to maintain long-term efficacy.

Future research should expand beyond a single-city, single-disease focus to include multi-regional and multi-disease analyses, integrate control groups where feasible, and explore the behavioral responses of providers and patients over longer time horizons. Such efforts will contribute to a more comprehensive understanding of the dynamic impacts of DRG reform and inform evidence-based policy decisions in China and other developing health systems undergoing similar transitions.

## 6. Conclusions

This study utilizes a natural experiment from a 2022 DRG payment reform in a major Chinese city to evaluate its impact on healthcare quality and efficiency using inpatient data from colorectal cancer patients (2020–2023). Our study indicates that the initial stage of DRG implementation achieved its preliminary goals of optimizing medical resource allocation and improving the efficiency of medical insurance fund utilization. However, the reform still needs continuous policy monitoring, long-term effectiveness evaluation, and adaptive strategies tailored to heterogeneous institutional contexts. For future DRG reform in China, policymakers should consider strategies to mitigate the financial burden on patients while sustaining cost-control incentives for providers. Differentiated policy designs may be necessary across insurance types and hospital tiers.

## Figures and Tables

**Table 1:** Key dependent, independent, and control variables.

	Variable Name	Variable Measurement
Dependent variables	Total Expenditure	Total hospitalization costs.
	Medical Costs Paid by Medical Insurance	The portion of costs reimbursed by the Urban Employee Basic Medical Insurance (UEBMI) or Urban-Rural Resident Basic Medical Insurance (URRBMI) schemes.
	Out-of-Pocket	The amount paid directly by patients.
	Length of Stay (LOS)	The total number of days a patient spent in the hospital during one admission. Used as a proxy for healthcare efficiency.
	30-day Readmission Rate	A binary indicator equal to 1 if the patient was readmitted for any cause within 30 days of discharge, and 0 otherwise.
	Low-risk mortality Rate	Mortality among patients classified as “low-risk” (pathological stage l- II and Charlson Comorbidity Index ≤ 1).
	Medicine Costs	The total medicine costs incurred.
	Diagnostic Examination Costs	The total diagnostic costs incurred.
	Medical Consumable Costs	The total medical consumable costs incurred.
Control variables	Age	The age of patient
	Gender	female = 0, male = 1
	Hospital Type	General hospital, specialty hospital, traditional Chinese medicine hospital, etc.
	Classification of Hospital (COH)	Classification of hospitals in China (e.g., secondary vs. tertiary; Grade A is the highest accreditation level).
	Type of Medical Insurance	UEBMI (urban employee) vs. URRBMI (urban and rural resident). UURBMI = 0, UEBMI = 1
	Charlson Comorbidity Index (CCI)	A weighted score of comorbidities, adjusted for age, where higher values indicate greater disease severity.

**Table 2:** Descriptive statistics of variables.

Variables		N	Mean/Rate	SD
Total expenditure (CNY)		66,533	16,592	17,736
Medical costs paid by medical insurance (CNY)		66,533	12,347	12,669
out-of-pocket (CNY)		66,533	4245	6394
Length-of-stay (LOS)		66,533	6.66	7.16
30-day readmission plan rate		66,533	0.69	0.02
Low-risk mortality rate		66,533	0.10	0.05
out-of-pocket rate		66,533	0.23	0.13
Medicine costs (CNY)		66,509	7922	7817
Diagnostic examination costs (CNY)		66,509	6178	7494
Medical consumable costs (CNY)		66,509	2491	5997
Age		66,533	65.17	11.05
CCI		66,533	2.10	1.06
Gender	Male	41,708	63%	-
	Female	24,825	37%	-
Medical insurance type	UEBMI	51,285	77%	-
	URRBMI	15,248	23%	-
Different classification of hospital (COH)	Tertiary hospital	56,994	87%	-
	Secondary hospital	8190	13%	-
Hospitals type	Specialty Hospital	9015	13.82%	-
	Traditional Chinese Medicine Hospital	14,764	22.64%	-
	Community Health Center	261	0.40%	-
	Integrated Chinese-Western Medicine Hospital	1435	2.20%	-
	General Hospital	39,716	60.89%	-
	Maternal and Child Health Hospital	35	0.05%	-
Tertiary Grade-A hospital	Grade-A	52,438	80%	-
	Not Grade-A	12,790	20%	-

**Table 3:** Significant difference in key indicators before and after reform.

Variables	Before		After		Difference
	N1	Mean1	N2	Mean2	
Total expenditure (CNY)	24,692	16,499	41,841	16,647	147.90
Medical costs paid by medical insurance (CNY)	24,692	12,731	41,841	12,120	−610.54 ***
out-of-pocket (CNY)	24,692	3768	41,841	4526	758.44 ***
LOS	24,692	7.11	41,841	6.40	−0.71 ***
30-day readmission plan rate	24,692	0.71	41,841	0.68	−0.03 ***
Low-risk mortality rate	24,692	0.16	41,841	0.07	−0.09 ***
Out-of-pocket rate	24,692	0.22	41,841	0.24	0.02 ***
Medicine costs (CNY)	24,670	9037	41,839	7264	−1773.14 ***
Diagnostic examination costs (CNY)	24,670	5441	41,839	6614	1172.95 ***
Medical consumable costs (CNY)	24,670	2020	41,839	2769	749.42 ***
CCI	24,692	2.14	41,841	2.07	−0.07 ***

Note: *** *p* < 0.01.

**Table 4:** The Impact of DRG reform on medical costs and efficiency.

Variables	LOS	Total Expenditure	Medical Costs Paid by Medical Insurance	Out-of-Pocket Rate	Medicine Costs	Diagnostic Examination Costs	Medical Consumable Costs
Effects	−2.06 ***	−0.13 **	−0.25 ***	0.08 ***	−0.04	−0.23 **	−0.20
	(−3.43, −0.70)	(−0.26, −0.00)	(−0.39, −0.12)	(0.05, 0.10)	(−0.34, 0.25)	(−0.41, −0.05)	(−0.45, 0.05)
Observations	65,184	65,184	65,058	65,184	65,076	65,161	65,116

Note: *** *p* < 0.01, ** *p* < 0.05.

**Table 5:** The Impact of DRG reform on healthcare quality.

Variables	30-Day Readmission Plan Rate	Low-Risk Mortality Rate	CCI
Effects	−0.01 ***	−0.04 ***	0.01
	(−0.01, −0.00)	(−0.04, −0.03)	(−0.03, 0.04)
Observations	65,184	65,184	65,184

Note: *** *p* < 0.01.

**Table 6:** Comparison of different insurance types before and after the reform.

Variables	LOS	Total Expenditure	Medical Costs Paid by Medical Insurance	Out-of-Pocket Rate	30-Day Readmission Plan Rate	Low-Risk Mortality Rate	Medicine Costs	Diagnostic Examination Costs	Medical Consumable Costs
UEBMI									
Effects	−1.87 ***	−0.12 *	−0.21 ***	0.06 ***	−0.01 ***	−0.03 ***	−0.01	−0.24 **	−0.21
	(−3.22, −0.53)	(−0.25, 0.02)	(−0.35, −0.07)	(0.03, 0.09)	(−0.01, −0.00)	(−3.61, −3.01)	(−0.33, 0.30)	(−0.42, −0.05)	(−0.47, 0.05)
Observations	50,366	50,366	50,245	50,366	50,366	50,366	50,287	50,347	50,314
URRBMI									
Effects	−2.21 **	−0.15	−0.37 ***	0.15 ***	−0.01 ***	−0.04 ***	−0.09	−0.21 *	−0.34 *
	(−3.95, −0.47)	(−0.33, 0.03)	(−0.58, −0.16)	(0.11, 0.18)	(−0.01, −0.00)	(−4.34, −3.70)	(0–0.41, 0.22)	(−0.43, 0.01)	(−0.7, 0.01)
Observations	14,818	14,818	14,813	14,818	14,818	14,818	14,789	14,814	14,802

Note: *** *p* < 0.01, ** *p* < 0.05, * *p* < 0.1.

**Table 7:** Comparison of COH before and after the reform.

Variables	LOS	Total Expenditure	Medical Costs Paid by Medical Insurance	Out-of-Pocket Rate	30-Day Readmission Plan Rate	Low-Risk Mortality Rate	Medicine Costs	Diagnostic Examination Costs	Medical Consumable Costs
Tertiary hospital									
Effects	−1.75 **	−0.14 **	−0.25 ***	0.08 ***	−0.01 ***	−3.47 ***	−0.05	−0.23 **	−0.19
	(−3.12, −0.37)	(−0.28, −0.00)	(−0.40, −0.11)	(0.05, 0.11)	(−0.01, −0.00)	(−3.77, −3.17)	(−0.38, 0.27)	(−0.43, −0.04)	(−0.46, 0.07)
Observations	56,994	56,994	56,873	56,994	56,994	56,994	56,900	56,971	56,936
Secondary hospital									
Effects	−3.18 **	−0.13	−0.20	0.06 ***	−0.01 ***	−4.01 ***	0.13	−0.30 **	−3.18 **
	(−6.06, −0.29)	(−0.53, 0.26)	(−0.60, 0.21)	(0.02, 0.09)	(−0.01, −0.00)	(−4.34, −3.68	(−0.36, 0.61)	(−0.54, −0.05)	(−1.06, 0.35)
Observations	8190	8190	8185	8190	8190	8190	8176	8190	8190
Tertiary Grade-A hospital									
Effects	−1.43 **	−0.13 *	−0.25 ***	0.08 ***	−0.01 ***	−3.48 ***	−0.05	−0.22 **	−0.23
	(−2.82, −0.04)	(−0.28, 0.02)	(−0.41, −0.08)	(0.05, 0.11)	(−0.01, −0.00)	(−3.80, −3.17)	(−0.41, 0.30)	(−0.43, −0.01)	(−0.52, 0.06)
Observations	52,438	52,438	52,324	52,438	52,438	52,438	52,350	52,417	52,383

Note: *** *p* < 0.01, ** *p* < 0.05, * *p* < 0.1.

**Table 8:** Continuity test of control variables.

Variables	LOS	Total Expenditure	Medical Costs Paid by Medical Insurance	Low-Risk Mortality
Gender	0.04	−0.03	0.06	0.01
	(0.11)	(0.18)	(0.22)	(0.13)
Age	0.69	2.18	2.4084	1.84
	(2.27)	(3.36)	(4.10)	(2.69)
CCI	−0.05	0.01	−0.01	0.01
	(0.22)	(0.33)	(0.41)	(0.25)
Hospitals type	−0.36	−0.51	−0.66	−0.51
	(0.35)	(0.56)	(0.69)	(0.40)
COH	0.01	0.03	0.09	−0.01
	(0.07)	(0.11)	(0.12)	(0.08)
Medical insurance type	0.13	0.24	0.34 *	0.18 *
	(0.10)	(0.17)	(0.20)	(0.11)
Statistic	0.40	−0.29	−0.50 *	−0.40
	(1.18)	(0.23)	(0.30)	(0.36)
Observations	65,184	65,184	65,184	65,184

Note: * *p* < 0.1, Standard errors in parentheses.

**Table 9:** Robustness Check: Bandwidth Sensitivity Analysis.

Variables	LOS	Total Expenditure	Medical Costs Paid by Medical Insurance	Out-of-Pocket Rate	30-Day Readmission Plan Rate	Low-Risk Mortality Rate	Medicine Costs	Diagnostic Examination Costs	Medical Consumable Costs
0.8									
Effects	−2.16 ***	−0.12 *	−0.26 ***	0.07 ***	−0.01 ***	−0.03 ***	−0.02	−0.23 **	−0.21 *
	(−3.65, −0.67)	(−0.25, 0.01)	(−0.40, −0.12)	(0.04, 0.10)	(−0.01, −0.00)	(−3.72, −3.11)	(−0.32, 0.29)	(−0.42, −0.04)	(−0.45, 0.03)
1.2									
Effects	−1.79 ***	−0.14 **	−0.26 ***	0.08 ***	−0.01 ***	−0.04 ***	−0.06	−0.22 **	−0.22 *
	(−3.04, −0.53)	(−0.27, −0.02)	(−0.40, −0.12)	(0.06, 0.11)	(−0.01, −0.00)	(−3.83, −3.33)	(−0.35, 0.23)	(−0.39, −0.05)	(−0.47, 0.02)
Observations	65,184	65,184	65,058	65,184	65,184	65,184	65,076	65,161	65,116

Note: *** *p* < 0.01, ** *p* < 0.05, * *p* < 0.1.

**Table 10:** Parameter Estimates of DRG Reform Effects on Outcomes.

Variables	LOS	Total Expenditure	Medical Costs Paid by Medical Insurance	Out-of-Pocket Rate	30-Day Readmission Plan Rate	Low-Risk Mortality Rate	Medicine Costs	Diagnostic Examination Costs	Medical Consumable Costs
Effects	−1.61 ***	−0.11	−0.24 ***	0.08 ***	−0.01 ***	−0.04 ***	−0.09	−0.22 **	−0.18
	(−2.81, −0.40)	(−0.24, 0.03)	(−0.04, −0.10)	(0.05, 0.10)	(−0.01, −0.00)	(−0.04, −0.03)	(−0.38, 0.21)	(−0.41, −0.02)	(−0.44, 0.07)
Observations	65,184	65,184	65,058	65,184	65,184	65,184	65,076	65,161	65,116

Note: *** *p* < 0.01, ** *p* < 0.05.

**Table 11:** The Effects of DRG Reform Without Controls.

Variables	LOS	Total Expenditure	Medical Costs Paid by Medical Insurance	Out-of-Pocket Rate	30-Day Readmission Plan Rate	Low-Risk Mortality Rate	Medicine Costs	Diagnostic Examination Costs	Medical Consumable Costs
Effects	−1.81 **	−0.13 *	−0.24 ***	0.08 ***	−0.01 ***	−0.03 ***	−0.02	−0.24 ***	−0.20
	(−3.28, −0.34)	(−0.28, 0.02)	(−0.38, −0.09)	(0.05, 0.11)	(−0.01, −0.00)	(−0.04, −0.03)	(−0.34, 0.30)	(−0.41, −0.06)	(−0.47, 0.07)
Observations	66,533	66,533	66,406	66,533	66,533	66,533	66,403	66,507	66,441

Note: *** *p* < 0.01, ** *p* < 0.05, * *p* < 0.1.

**Table 12:** Placebo Test of DRG Reform Effects.

Variables	LOS	Total Expenditure	Medical Costs Paid by Medical Insurance	Out-of-Pocket Rate	30-Day Readmission Plan Rate	Low-Risk Mortality Rate	Medicine Costs	Diagnostic Examination Costs	Medical Consumable Costs
Effects	0.01	−0.01	0.01	−0.01	−0.01 ***	−0.37 ***	0.07	−0.06	−0.03
	(−1.14, 1.17)	(−0.11, 0.11)	(−0.11, 0.12)	(−0.04, 0.04)	(−0.01, −0.00)	(−0.45, −0.30)	(−0.09, 0.23)	(−0.24, 0.12)	(−0.31, 0.24)
Observations	65,184	65,184	65,058	65,184	65,184	65,184	65,076	65,161	65,116

Note: *** *p* < 0.01.

**Table 13:** Effects of DRG Reform after Excluding COVID-19 Period.

Variables	LOS	Total Expenditure	Medical Costs Paid by Medical Insurance	Out-of-Pocket Rate	Medicine Costs	Diagnostic Examination Costs	Medical Consumable Costs	30-Day Readmission Plan Rate	Low-Risk Mortality Rate	CCI
Effects	−2.18 ***	−0.16 **	−0.26 ***	0.07 ***	−0.09	−0.26 ***	−0.22 *	−0.01 ***	−3.61 ***	0.02
	(−3.64, −0.72)	(−0.30, −0.01)	(−0.41, −0.12)	(0.04, 0.10)	(−0.45, 0.26)	(−0.43, −0.08)	(−0.45, 0.02)	(−0.01, −0.00)	(−3.85, −3.36)	(−0.02, 0.06)
Observations	54,425	54,425	54,322	54,425	54,339	54,407	54,375	54,425	54,425	54,425

Note: *** *p* < 0.01, ** *p* < 0.05, * *p* < 0.1.

## Data Availability

Data are contained within the article.

## Appendix A. Construction of Age-Adjusted Charlson Comorbidity Index

**Table:**

Comorbidities	Weight	ICD-10
Myocardial infarction.	1	I21|I22|I25.2
Congestive heart failure.	1	I09.9|I11.0|I13.2|I13.0|I25.5|I42.0|I42.5|II42.6|I42.7|I42.8|I42.9|I43|I50|P29.0
Peripheral vascular disease.	1	I70|I71|I73.1|I73.8|I73.9|I77.1|I79.0|I79.2|K55.1|K55.8|K55.9|Z95.8|Z95.9
Cerebrovascular disease.	1	G45|G46|H34.0|I60|I61|I62|I63|I64|I65|I66|I67|I68|I69
Dementia.	1	F00|F01|F02|F03|F05.1|G30|G31.1
Chronic pulmonary disease.	1	J40|J41|J42|J43|J44|J45|J46|J47|I27.8|I27.9|J60|J61|J62|J63|J64|J64|J66|J67|J68.4|J70.1|J70.3
Rheumatic disease.	1	M05|M06|M32|M33|M34|M35|M31.5|M35.1|M35.3|M36.0
Peptic ulcer disease.	1	K25|K26|K27|K28
Mild liver disease.	1	B18|K70.0|K70.1|K70.2|K70.3|K70.9|K71.3|K71.4|K71.5|K71.7|K73|K74|K76.0|K76.2|K76.3|K76.4|K76.8|K76.9|Z94.4
Diabetes without chronic complication.	1	E10.0|E10.1|E10.6|E10.8|E10.9|E11.0|E11.1|E11.6|E11.8|E11.9|E12.0|E12.1|E12.6|E12.8|E12.9|E13.0|E13.1|E13.6|E13.8|E13.9|E14.0|E14.1|E14.6|E14.8|E14.9
Diabetes with chronic complication.	2	E10.2|E10.3|E10.4|E10.5|E10.7|E11.2|E11.3|E11.4|E11.5|E11.7|E12.2|E12.3|E12.4|E12.5|E12.7|E13.2|E13.3|E13.4|E13.5|E13.7|E14.2|E14.3|E14.4|E14.5|E14.7
Hemiplegia or paraplegia.	2	G04.1|G11.4|G80.1|G80.2|G81|G82|G83.0|G83.1|G83.2|G83.3|G83.4|G83.9
Renal disease.	2	I12.0|I13.1|N03.2|N03.3|N03.4|N03.5|N03.6|N03.7|N05.2|N05.3|N05.4|N05.5|N05.6|N05.7|N18|N19|N25.0|Z49.0|Z49.1|Z49.2|Z94.0|Z99.2
Any maliqnancy, including lymphoma and leukemia, except malignant neoplasm of skin.	2	C00|C01|C02|C03|C04|C05|C06|C07|C08|C09|C10|C11|C12|C13|C14|C15|C16|C17|C18|C19|C20|C21|C22|C23|C24|C25|C26|C30|C31|C32|C33|C34|C37|C38|C39|C40|C41|C43|C45|C46|C47|C48|C49|C50|C51|C52|C53|C54|C55|C56|C57|C58|C60|C61|C62|C63|C64|C65|C66|C67|C68|C69|C70|C71|C72|C73|C74|C75|C76|C81|C82|C83|C84|C85|C88|C90|C91|C92|C93|C94|C95|C96|C97
Moderate or severe liver disease.	3	I85.0|I85.9|I86.4|I98.2|K70.4|K71.1|K72.1|K72.9|K76.5|K76.6|K76.7
Metastatic solid tumor.	6	C77|C78|C79|C80
AIDS/HIV.	6	B20|B21|B22|B24
Age-adjusted	Weight	---
<50	0	---
[50, 59]	1	---
[60, 69]	2	---
[70, 79]	3	---
[80, 80+]	4	---
